# Vision-Based Corrosion Detection Assisted by a Micro-Aerial Vehicle in a Vessel Inspection Application

**DOI:** 10.3390/s16122118

**Published:** 2016-12-14

**Authors:** Alberto Ortiz, Francisco Bonnin-Pascual, Emilio Garcia-Fidalgo, Joan P. Company-Corcoles

**Affiliations:** Department of Mathematics and Computer Science, University of Balearic Islands, Palma de Mallorca 07122, Spain; xisco.bonnin@uib.es (F.B.-P.); emilio.garcia@uib.es (E.G.-F.); joanpep.company@uib.es (J.P.C.-C.)

**Keywords:** vessel inspection, defect detection, unmanned aerial vehicle, supervised autonomy, machine learning, artificial neural network

## Abstract

Vessel maintenance requires periodic visual inspection of the hull in order to detect typical defective situations of steel structures such as, among others, coating breakdown and corrosion. These inspections are typically performed by well-trained surveyors at great cost because of the need for providing access means (e.g., scaffolding and/or cherry pickers) that allow the inspector to be at arm’s reach from the structure under inspection. This paper describes a defect detection approach comprising a micro-aerial vehicle which is used to collect images from the surfaces under inspection, particularly focusing on remote areas where the surveyor has no visual access, and a coating breakdown/corrosion detector based on a three-layer feed-forward artificial neural network. As it is discussed in the paper, the success of the inspection process depends not only on the defect detection software but also on a number of assistance functions provided by the control architecture of the aerial platform, whose aim is to improve picture quality. Both aspects of the work are described along the different sections of the paper, as well as the classification performance attained.

## 1. Introduction

The different steel surfaces that are part of a vessel’s hull can be affected by different kinds of defective situations, such as coating breakdown, corrosion, and, ultimately, cracks. These defects are indicators of the state of the metallic surface and, as such, an early detection prevents the structure from buckling or fracturing, and, ultimately, the personal, environmental and financial catastrophic consequences this can give rise to. To avoid reaching such undesirable situations, inspections onboard sea-going vessels are regular activities being initiated partly due to applicable classification and statutory regulations, and partly because of the obvious interest of ship operators and ship owners in anticipating the defective situations, for safety reasons but also because of the costs associated to unexpected disruptions of vessel service availability.

To carry out this task, the vessel has to be emptied and situated in a dockyard where scaffolding and/or cherry-pickers must be used to allow the human inspectors to reach the areas under inspection. For some vessels (e.g., Ultra Large Crude Carriers, ULCC), this process can mean the visual assessment of more than 600,000 m^2^ of steel. Total expenses required for this kind of close-up inspection can reach up to $1M once you factor in the use of yard’s facilities and the vessel’s preparation, i.e., cleaning, ventilation, and provision of access arrangements. Consequently, since visual inspections are and will be an important source of information for structure condition assessment, it is clear that the introduction of new technological tools will lead to significant reductions of the effort and costs related to inspections.

In this regard, one of the main goals of the already concluded EU FP7 project MINOAS [1] was to develop a fleet of robotic platforms with different locomotion capabilities with the aim of teleporting the human surveyor to the different vessel structures to be inspected. Given the enormity of these structures and the requirement for vertical motion as part of the inspection process, a multi-rotor platform belonging to the Micro-Aerial Vehicles (MAVs) class was selected as one of the members of the fleet due to their small size, agility and fast deployment time (see the work by Bonnin-Pascual et al. [2]). In accordance to some constructive advice from end-users at the end of project MINOAS (see the work by Eich et al. [3]), this platform has been under re-design within the EU FP7 follow-up project INCASS [4], a first version of which was described in Bonnin-Pascual et al. [5].

This paper presents a novel solution for detecting coating breakdown/corrosion (CBC) as a support for surveyors during visual inspections of vessels. The solution here described adopts an approach based on a semi-autonomous MAV fitted with functionalities intended to enhance image capture by means of extensive use of behaviour-based high-level control, and an *artificial neural network* (ANN) which discriminates between pixels suspected/not suspected to correspond to CBC-affected areas by means of adequate colour and texture descriptors. A first version of the neural network-based detector, employing different descriptors, was described in Ortiz et al. [6]. Here we present an enhanced network, which, in turn, has been submitted to more extensive testing.

By way of summary of this paper contributions: (1) we address defect detection in vessels by means of an assistant robot; (2) we describe a control architecture specifically developed to improve visual inspection and, by extension, image capture, to enhance subsequent processing steps performance; (3) we propose new colour and texture descriptors for CBC detection; (4) we design the classifier with the only restriction of being an ANN, in order not to miss any useful configuration of the network, what means that, among others, we consider a number of different configurations of the CBC descriptor varying the involved parameters, as well as alternative CBC descriptors; (5) we evaluate the detector using a varied set of images taken under different conditions (hence irrespective of the platform capabilities as for image capture); and (6) finally we report results for a set of images taken during field trials, in a real vessel, which took place in recent dates, and using the particular capabilities of the robot.

The rest of the paper is organized as follows: Section 2 describes the inspection problem and outlines related work, Section 3 gives the details of the aerial platform, Section 4 outlines the defect detection approach, Section 5 configures the CBC detector and reports on detection performance, and, finally, Section 6 concludes the paper and outlines future work.

## 2. Background and Related Work

### 2.1. Inspection Problem and Platform Requirements

To perform a complete hull inspection, the vessel has to be emptied and situated in a dockyard, where typically temporary staging, lifts, movable platforms, etc., need to be installed to allow the workers to carry out close-up inspection—i.e., at the reach of a hand—of the different metallic surfaces and structures. For those ships where there is a real cost saving, i.e., the inspection is likely to result in no repair, so that the preparation of the vessel for a human inspection with a non-subsequent repair is less justified (see the work by Ortiz et al. [7] for a deeper analysis), robotic platforms can replace the in-situ human inspection.

**Figure 1:**
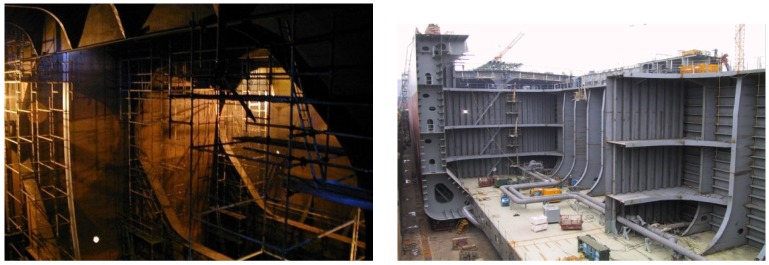
(**Left**) Staging required during a vessel inspection; (**Right**) Oil tanker in shipyard during construction.

Among others, the vertical structures that can be found in vessel holds are of prime importance (see Figure 1). To make proper repair/no repair decisions, the surveyor must be provided with, among others, imagery detailed enough so as to enable the remote visual assessment of these structures. The INCASS aerial platform is precisely intended to provide this kind of data during and after an inspection operation. To this end, the platform can be either required to sweep the relevant metallic surfaces and grab pictures at a rate compatible with its speed, or else provide visual evidence of the state of a particular area suspected of being defective. Those images must also be tagged with pose information, so that images suspected to contain defects can be associated to vessel structures, or even be compared across inspections when they correspond to the same structural element.

Therefore, the main requirements for the aerial platform stem directly from the very nature of the inspection process: the vehicle must be able to perform vertical, stationary and low speed flight, as well as permit indoor flight. These requirements rapidly discard fixed-wing aircrafts and focus the search on helicopter-type UAVs, naturally capable of manoeuvres such as hovering and vertical take-off and landing (VTOL). Additionally, the platform should not rely on GPS data for positioning because it could be required to operate indoors or in poor GPS reception areas (e.g., due to satellites being occluded by the vessel structures, multi-path effects, etc.).

A final requirement comes from the end-users, which during the field trials at the end of the preceding project MINOAS suggested the implementation of a friendly, flexible and robust way to interact with the platform so that they could take the robot to any point of a cargo hold without the need to be an expert pilot (instead of the approach based on way-point navigation adopted in MINOAS [2,3], which required the specification of a precise list of points for each mission, what meant an unnecessary rigidity when defining inspection operations).

### 2.2. Aerial Robots for Visual Inspection

Multi-rotor platforms have become increasingly popular in recent years, and, as a consequence, a number of control and navigation solutions—including platform stabilization, self-localization, mapping, and obstacle avoidance—can be found in the related literature. They mainly differ in the navigation sensor suite, the amount of processing that is performed onboard/off-board, and the assumptions made about the environment.

For a start, the laser scanner has been extensively used due to its accuracy and speed. For instance, Dryanovski et al. [8] and Grzonka et al. [9] propose full navigation systems using laser scan matching and IMU fusion for motion estimation embedded within SLAM frameworks that enable MAVs to operate indoors. Bachrach et al. [10] describe a laser-based multi-level approach for 3D mapping tasks, as well as Dryanovski et al. [8].

Infrared or ultrasound sensors are other possibilities for implementing navigation solutions. Although they typically have less accuracy and require higher noise tolerance, several researchers have used them to perform navigation tasks in indoor environments as an option cheaper than laser scanners, e.g., see the works by Bouabdallah et al. [11], Matsue et al. [12] and Roberts et al. [13].

Vision cameras have also been under consideration lately. Cameras’ success in general robotics comes mainly from the richness of the sensor data supplied, combined with their low weight, low power designs, and relatively low prices after the irruption of imaging CMOS technology. For the particular case of MAVs, the higher computational cost associated to vision-based navigation has led researchers to find optimized solutions that can run over low-power processors. Among the most recent papers published in this regard, some propose visual SLAM solutions based on feature tracking, either adopting a frontal mono or stereo camera configuration, e.g., Engel et al. [14] or Fraundorfer et al. [15], or choosing a ground-looking orientation, e.g., Chowdhary et al. [16]. Others focus on efficient implementations of optical flow calculations, either dense or sparse, and mostly from ground-looking cameras, e.g., Zingg et al. [17], or develop methods for landing, tracking and taking off using passive, e.g., Meier et al. [18], or active markers, e.g., Wenzel et al. [19], also adopting a ground-looking configuration.

Some of the aforementioned developments have resulted in a number of aerial robots-based approaches addressing inspection field problems. On the one hand, Huerzeler et al. [20] describe some scenarios for industrial and generic visual inspection using aerial vehicles, discussing as well the platforms’ requirements. In coincidence with part of the requirements outlined above for vessel inspection, the authors highlight the fact that inspections are usually performed in GPS-denied environments where motion tracking systems can not be installed. For this reason, aerial platforms for inspection must estimate their own state (attitude, velocity and/or position) relying on inner sensors and typically using onboard computational resources. As mentioned above, some approaches fuse visual (typically stereo) and inertial data to estimate the vehicle state, e.g., Burri et al. [21] or Omari et al. [22], while some others make use of laser range finders for positioning and mapping and the camera is only used for image capture, e.g., Bonnin-Pascual et al. [2] or Satler et al. [23]. Finally, some contributions rely on the specific configuration of the element under inspection, such as the approach described in Sa et al. [24], which is intended for the inspection of pole-like structures.

### 2.3. Defect Detection

Referring to automated vision-based defect detection, the scientific literature contains an important number of proposals. Among other possibilities, these can be roughly classified in two categories, depending on whether they look for defects specific of particular objects or surfaces, e.g., LCD displays by Chang et al. [25], printed circuit boards by Jiang et al. [26], copper strips by Zhang et al. [27], ceramic tiles by Boukouvalas et al. [28], etc., or, to the contrary, they aim at detecting general and unspecific defects, e.g., see the works by Amano [29], Bonnin-Pascual and Ortiz [30], Castilho et al. [31], Hongbin et al. [32], and Kumar and Shen [33].

Within the first category (which would also involve our approach for corrosion detection), one can find a large collection of contributions for automatic vision-based crack detection, e.g., for concrete surfaces see the works by Fujita et al. [34], Oulette et al. [35], Yamaguchi and Hashimoto [36] and Zhao et al. [37], for airplanes see the work by Mumtaz et al. [38], etc. However, regarding corrosion, to the best of our knowledge, the number of works which can be found is rather reduced [38,39,40,41,42,43]. First of all, Jahanshahi and Masri [39] make use of colour wavelet-based texture analysis algorithms for detecting corrosion, while Ji et al. [40] utilize the watershed transform applied over the gradient of gray-level images, Siegel et al. [41] use wavelets for characterizing and detect corrosion texture in airplanes, Xu and Weng [42] adopt an approach based on the fractal properties of corroded surfaces and Zaidan et al. [43] also focus on corrosion texture using the standard deviation and the entropy as discriminating features.

## 3. The Aerial Platform

This section describes the aerial platform which takes the pictures which will be lately processed for CBC detection. This platform in turn provides the localization information which is associated with every picture, in order to better locate the defect over the vessel structures.

### 3.1. General Overview

**Figure 2:**
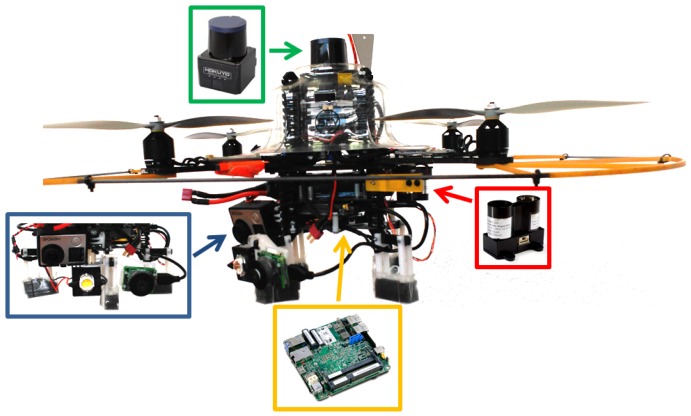
A realization of the INCASS aerial platform: (**green**) laser scanner; (**red**) height sensor; (**yellow**) embedded PC and (**blue**) camera set and illumination.

The aerial platform comprises a multi-rotor vehicle fitted with a *flight management unit* (FMU) for platform stabilization in roll, pitch and yaw, and thrust control, a 3-axis *inertial measuring unit* (IMU)—which, according to today standards, is typically part of the FMU—a sensor suite able to supply vehicle 3D speed and height measurements, as well as distances to surrounding obstacles, and an embedded PC which avoids sending sensor data to a base station, but process them onboard and, thus, prevent communications latency inside critical control loops. Figure 2 shows a realization of this platform taking as a base the Pelican quadrotor by Ascending Technologies.

As it is well known, the AscTec FMU is equipped with a 3-axis gyroscope, a 3-axis accelerometer and a 3-axis magnetometer, together with two ARM7 microcontrollers, one implementing the FMU (and hence running the platform firmware) and the other reserved to higher-level control loops which can be programmed by the user. Apart from this, the realization of Figure 2 features:

- The lightweight laser scanner Hokuyo UST-20LX, which provides 20 m coverage for a 270^∘^ angular sector. This sensor is used to estimate 2D speed as well as distances to the surrounding obstacles.
- A downward-looking LIDAR-Lite laser range finder used to supply height data for a maximum range of 40 m. Vertical speed is estimated by proper differentiation of the height measurements.
- Two cameras to collect, from the vessel structures under inspection, images on demand (a Chameleon 3 camera, by Pointgrey (Richmond, VA, USA), fitted with a Sony IMX265, CMOS, 1/1.8^″^, 2048 × 1536-pixel imaging sensor, by Sony (Tokyo, Japan), and a fixed-focal length lightweight M12 8 mm lens) and video footage (a GoPro 4 camera, by Gopro (San Mateo, CA, USA), which supplies stabilized HD video).
- A 10 W pure white LED (5500–6500 K) delivering 130 lumens/watt for a 90^∘^ beam angle.
- An Intel NUC D54250WYB embedded PC featuring an Intel Core i5-4250U 1.3 GHz processor and 4 GB RAM.

**Figure 3:**
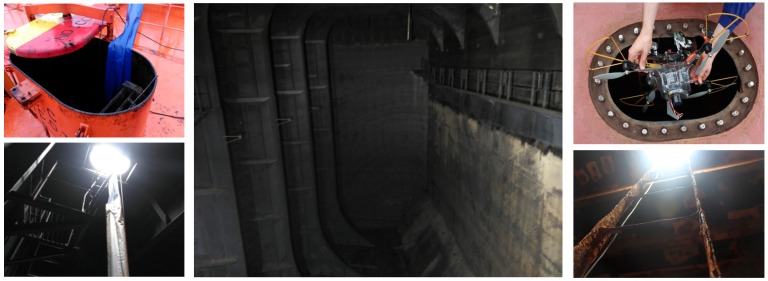
(**Left**) Oil tanker manhole entry point; (**Middle**) Typical oil tanker cargo hold; (**Right**) Entry point to a ballast tank of a bulk carrier.

Apart from other sensor suites capable of providing also speed and height measurements, the previous configuration allows operating under low-light conditions, as required in certain vessel compartments such as e.g., oil tanker cargo holds or, in general, ballast tanks, which are typically fitted with one single manhole-size entry point (approximately 600–800 × 600 mm, see Figure 3). In this regard, both cameras have been chosen because they are compatible with the payload restrictions and also for being able to produce useful images and video footage under low-light conditions, due to the imaging sensors and the underlying electronics they are fitted with.

### 3.2. Control Software

**Figure 4:**
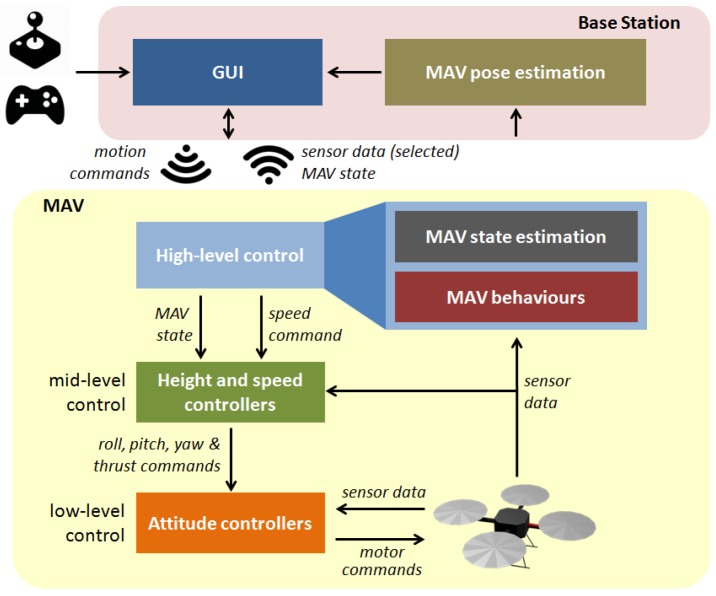
MAV software organization.

The aerial platform implements a control architecture that follows the *supervised autonomy* (SA) paradigm [44]. The control software is organized around a layered structure distributed among the available computational resources (see Figure 4). On the one hand, as said above, the *low-level control* layer implementing attitude stabilization and direct motor control is executed over the main microcontroller as the platform firmware provided by the manufacturer [45]. On the other hand, *mid-level control*, running over the secondary microcontroller, comprises height and speed controllers which map input speed commands into roll, pitch, yaw and thrust orders. The *high-level control* layer, which executes over the embedded PC, implements a reactive control strategy coded as a series of ROS nodes running over Linux Ubuntu, which combine the user desired speed command with the available sensor data—3-axis velocities $v_{x}$, $v_{y}$ and $v_{z}$, height *z* and distances to the closest obstacles $d_{i}$—to obtain a final and safe speed set-point that is sent to the speed controllers. Lastly, a *base station* (BS), also running ROS over Linux Ubuntu, linked with the MAV via a WiFi connection, executes the *human-machine interface* (HMI). The BS captures the user intention through the joystick/gamepad and sends the resulting qualitative commands to the MAV, supplies the operator with information about the state of the platform as well as about the task under execution through the GUI, and finally runs the self-localization strategy which, among others, is required to tag the images collected with the vehicle pose.

#### 3.2.1. Estimation of MAV State and Distance to Obstacles

**Figure 5:**
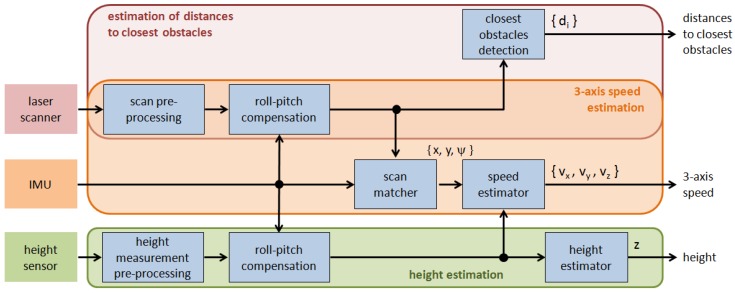
Estimation of Micro-Aerial Vehicles (MAV) state and distances to closest surrounding obstacles.

The platform state includes the vehicle velocities along the three axes, $v_{x}$, $v_{y}$ and $v_{z}$, and the flight height *z*. Apart from this, to compute the next motion orders, the control architecture requires the distances to the closest surrounding obstacles $d_{i}$. The estimation of all these values is performed by the corresponding three modules, as described in Figure 5. This figure also details the steps followed within each one of these modules for the particular case of the sensor configuration comprising one IMU, a laser scanner and a height sensor, as corresponds to the realization shown in Figure 2.

The estimation of 3-axis speed and the distances to closest obstacles share the *laser scan pre-processing* module (which essentially filters outliers) and the vehicle *roll and pitch compensation* module to obtain an ortho-projected scan on the basis of the IMU roll $\phi_{\mathrm{imu}}$ and pitch $\theta_{\mathrm{imu}}$ values. The processed scan is next used to both feed a *scan matcher*, which computes the platform 2D roto-translation between consecutive scans (x,y,ψ) using IMU yaw $\psi_{\mathrm{imu}}$ for initialization, and also to estimate distances to the closest surrounding obstacles $d_{i}$ (*closest obstacle detection* module), if any. The latter provides as many distances as angular subdivisions are made of the typically 270^∘^ angle range covered by the scanner. In our case, three sectors are considered, *front*, *left* and *right*, and the distances supplied are calculated as the minimum of all distances belonging to the corresponding sector. Finally, the *speed estimator* module determines 3-axis speed by means of a linear Kalman filter fed with the 2D translation vector (x,y) and the vehicle height *z*.

Regarding height estimation, after signal filtering (module *height measurement pre-processing*) and *roll-pitch compensation*, the processed height reaches the *height estimator* module, which, on the basis of the difference between two consecutive height measurements, decides whether this change is due to motion along the vertical axis or because of a discontinuity in the floor surface (e.g., the vehicle overflies a table).

#### 3.2.2. Generation of MAV Speed Commands

**Figure 6:**
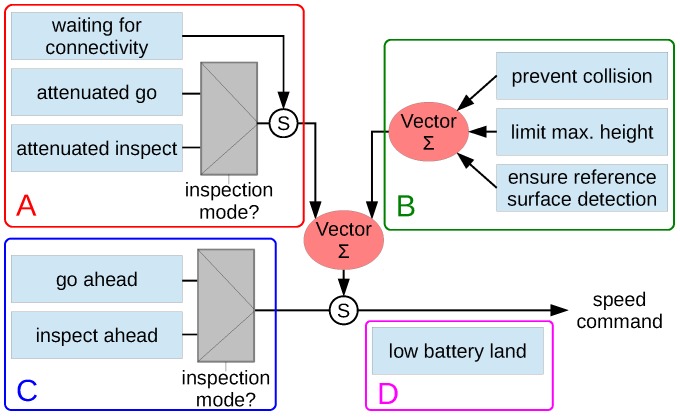
MAV behaviours: A—behaviours to accomplish the user intention; B—behaviours to ensure the platform safety within the environment; C—behaviours to increase the autonomy level; and D—behaviours to check flight viability.

Speed commands are generated through a set of robot behaviours organized in a hybrid competitive-cooperative framework [46]. The behaviour-based architecture is detailed in Figure 6, grouping the different behaviours depending on its purpose. A total of four general categories have been identified for the particular case of visual inspection: (a) *behaviours to accomplish the user intention*, which propagate the user desired speed command, attenuating it towards zero in the presence of close obstacles, or keeps hovering until the WiFi link is restored after an interruption; (b) *behaviours to ensure the platform safety within the environment*, which prevent the robot from colliding or getting off the safe area of operation, i.e., flying too high or too far from the reference surface that is involved in speed measurements; (c) *behaviours to increase the autonomy level*, which provide higher levels of autonomy to both simplify the vehicle operation and to introduce further assistance during inspections; and (d) *behaviours to check flight viability*, which checks whether the flight can start or progress at a certain moment in time. Some of the behaviours in groups (a) and (c) can operate in the so-called *inspection mode*. While in this mode, the vehicle moves at a constant and reduced speed (if it is not hovering) and user commands for longitudinal displacements or turning around the vertical axis are ignored. In this way, during an inspection, the platform keeps at a constant distance and orientation with regard to the front wall, for improved image capture.

#### 3.2.3. Base Station

**Figure 7:**
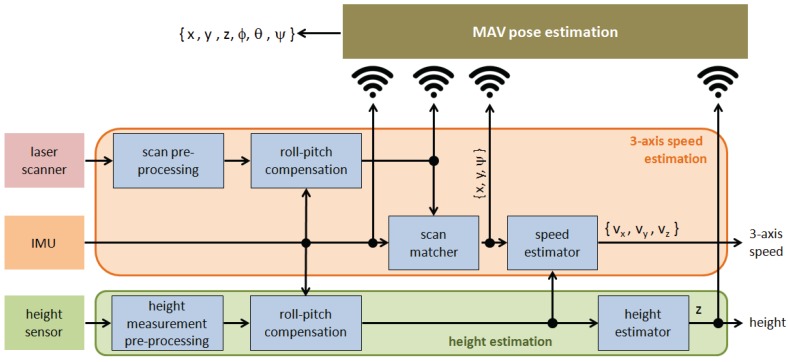
MAV pose estimation.

The BS runs the HMI, as mentioned before, as well as those processes that can tolerate communications latency, while critical control loops run onboard the vehicle in order to ensure minimum delay. One of the processes which run on the BS is the *MAV pose estimation* (see Figure 4 and Figure 7). Apart from being relevant by itself, the MAV pose is required to tag images with positioning information, so that they can be located over the vessel structure, as well as for comparing images across inspections. To this end, the BS collects pose data estimated by other modules under execution onboard the platform, height *z*, roll ϕ and pitch *θ*, and also runs a SLAM solution which counteracts the well-known drift that unavoidably takes place after some time of roto-translation integration. The SLAM module receives the projected laser scans and computes online a correction of the 2D subset (x,y,ψ) of the 6D robot pose (x,y,z,ϕ,θ,ψ), and a 2D map of the inspected area. We use the public ROS package *gmapping*, based on the work by Grisseti et al. [47], to provide the SLAM functionality.

## 4. Detection of Defects

This section describes a coating breakdown/corrosion (CBC) detector based on a three-layer perceptron configured as a *feed-forward neural network* (FFNN), which discriminates between the CBC and the NC (non-corrosion) classes.

### 4.1. Background

**Figure 8:**
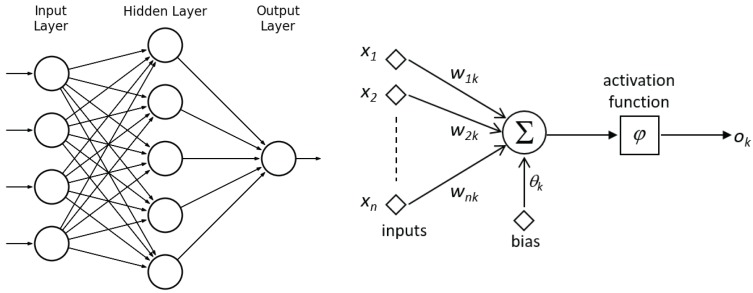
(**Left**) Topology of a feed-forward neural network (FFNN) comprising one single hidden layer; (**Right**) Structure of an artificial neuron.

An *artificial neural network* (ANN) is a computational paradigm that consists of a number of units (neurons) which are connected by weighted links (see Figure 8). This kind of computational structure learns from experience (rather than being explicitly programmed) and is inspired from the structure of biological neural networks and their way of encoding and solving problems. An FFNN is a class of ANN which organizes neurons in several layers, namely one input layer, one or more hidden layers, and one output layer, in such a way that connections exist from one layer to the next, never backwards [48], i.e., recurrent connections between neurons are not allowed. Arbitrary input patterns propagate forward through the network, finally causing an activation vector in the output layer. The entire network function, which maps input vectors onto output vectors, is determined by the connection weights of the net $w_{ij}$.

Every neuron *k* in the network is a simple processing unit that computes its activation output $o_{k}$ with respect to its incoming excitation $x=\{x_{i}\;|\;i=1,\cdots ,n\}$, in accordance to $o_{k}=\varphi \left(\sum_{i=1}^{n}w_{ik}x_{i}+\theta_{k}\right)$, where *φ* is the so-called activation function, which, among others, can take the form of, e.g., the hyperbolic tangent $\varphi \left(z\right)=2/\left(1+e^{-az}\right)-1$. Training consists in tuning weights $w_{ik}$ and bias $\theta_{k}$ mostly by optimizing the summed square error function $E=0.5\sum_{q=1}^{N}\sum_{j=1}^{r}{\left(o_{j}^{q}-t_{j}^{q}\right)}^{2}$, where *N* is the number of training input patterns, *r* is the number of neurons at the output layer and $\left(o_{j}^{q},t_{j}^{q}\right)$ are the current and expected outputs of the *j*-th output neuron for the *q*-th training pattern $x^{q}$. Taking as a basis the *back-propagation algorithm*, a number of alternative training approaches have been proposed through the years, such as the *delta-bar-delta rule*, *QuickpPop*, *Rprop*, etc. [49].

### 4.2. Network Features

**Figure 9:**
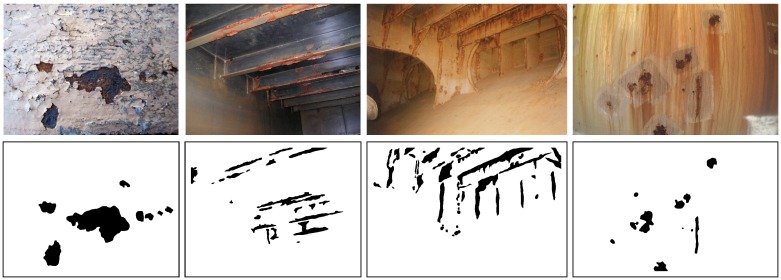
Examples of coating breakdown and corrosion: (**Top**) images from vessels, (**Bottom**) ground truth (pixels belonging to the coating breakdown/corrosion (CBC) class are labeled in black).

Figure 9 shows some examples of metallic structures affected by coating breakdown and/or corrosion. As can be expected, both colour and texture information are relevant for describing the CBC class. Accordingly, we define both colour and texture descriptors to characterize the neighbourhood of each pixel. Besides, in order to determine an optimal setup for the detector, we consider a number of plausible configurations of both descriptors and perform tests accordingly. Finally, different structures for the NN are considered varying the number of hidden neurons. In detail:

- For describing colour, we find the *dominant colours* inside a square patch of size ${\left(2w+1\right)}^{2}$ pixels, centered at the pixel under consideration. The colour descriptor comprises as many components as the number of *dominant colours* multiplied by the number of *colour channels*.
- Regarding texture, center-surround changes are accounted for in the form of *signed differences* between a central pixel and its neighbourhood at a given radius r(≠w) for every colour channel. The texture descriptor consists of a number of statistical measures about the differences occurring inside ${\left(2w+1\right)}^{2}$-pixel patches.
- As anticipated above, we perform a number of tests varying the different parameters involved in the computation of the patch descriptors, such as, e.g., the patch size *w*, the number of *dominant colours* *m*, or the size of the neighbourhood for *signed differences* computation (r,p).
- Finally, the number of hidden neurons hn are varied as a fraction *f* > 0 of the number of components *n* of the input patterns: hn=⌈f×n⌉.

The input patterns that feed the detector consist in the respective patch descriptors *D*, which result from stacking the texture and the colour descriptors, respectively $D_{\mathrm{texture}}$ and $D_{\mathrm{colour}}$:

(1) $$D=(D_{\mathrm{texture}},D_{\mathrm{colour}})$$

The details for both descriptors can be found in the following sections.

#### 4.2.1. Dominant Colours

**Figure 10:**
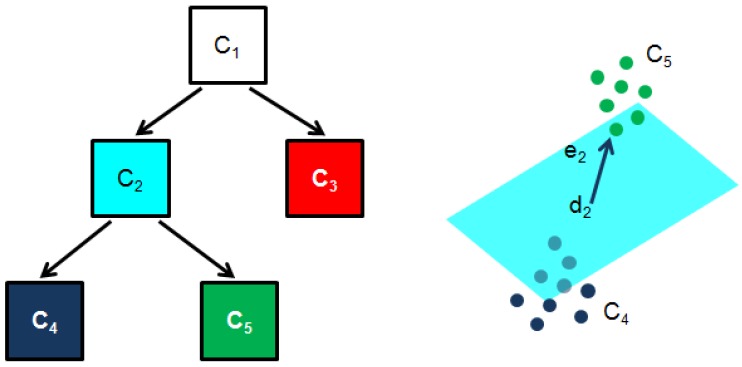
Illustration of the BIN *dominant colours* estimation method: 3 dominant colours result in this case; cluster $C_{2}$ splits into clusters $C_{4}=C_{2,L}$ and $C_{5}=C_{2,R}$ using the direction of largest colour variation $e_{2}$ and the average colour $d_{2}$.

The colour descriptor for a pixel results from quantizing the patch surrounding that pixel in a reduced number of representative colours, so called *dominant colours* (DC). In this work, we consider a binary-tree based clustering method attempting to minimize the *total squared error* (TSE) between the actual and the quantized patch. It is an adaptation of the algorithm described by Orchard and Bouman in [50], which we will refer to from now on as the BIN method. Briefly speaking, the clustering algorithm constrains the partitioning of the set of patch colours *C* to have the structure of a binary tree, whose nodes $C_{i}$ represent subsets of *C* and its two children split $C_{i}$ trying to minimize the TSE:

(2) $$\mathrm{TSE}=\sum_{d_{n}\in \mathrm{DC}}\sum_{j\in C_{n}}{∥c_{j}-d_{n}∥}^{2}\;,$$

where $d_{n}$ are the DC and $c_{j}$ are the colours belonging to $C_{n}$. The tree grows up until the number of tree leaves coincide with the number of desired DC (see Figure 10). Finally, node splitting is performed selecting the plane which bests separates the cluster colours. The algorithm chooses the plane whose normal vector is the direction of greatest colour variation and which contains the average colour $d_{i}$. As it is well known, this vector happens to be the eigenvector $e_{i}$ corresponding to the largest eigenvalue $\lambda_{i}$ of the node scatter matrix $\Sigma_{i}$:

(3) $$\sum_{j\in C_{i}}{\left({\left(c_{j}-d_{i}\right)}^{T}e_{i}\right)}^{2}=\lambda_{i}\;.$$

Colours at one side of the plane are placed in one of the node descendants $C_{i,R}$ and colours at the other side are placed in the other descendant $C_{i,L}$:

(4) $$C_{i,R}=\left\{j\in C_{i}\;s.t.\;e_{i}^{T}\left(c_{j}-d_{i}\right)\geq 0\right\}\;,\;C_{i,L}=\left\{j\in C_{i}\;s.t.\;e_{i}^{T}\left(c_{j}-d_{i}\right)<0\right\}\;.$$

At each stage of the algorithm, the leaf node with the largest eigenvalue is chosen for splitting. This strategy is not necessarily optimal, in the sense of the TSE, since it does not look ahead to the results of further splits, although it is expected to reduce the TSE proportionally to the total squared variation along the direction of the principal eigenvector, what performs well in general. Notice that the patch average colour is returned when only one DC is requested.

This clustering method has been chosen because of being simple although effective for our purposes. Other possibilities include the popular and well-known *k-means* [48], *NeuQuant* [51], *octree*-based [52] and *median cut* [53] quantizers.

Finally, to make more compact the features subspace spanned by the CBC class and thus make learning easier, the set of dominant colours is ordered in accordance to one of the colour channels, resorting to the other channels in case of tie. The colour descriptor is obtained stacking the requested *m* DC in the specified order:

(5) $$D_{\mathrm{colour}}=\left({\mathrm{DC}}_{1}^{\left(1\right)},{\mathrm{DC}}_{1}^{\left(2\right)},{\mathrm{DC}}_{1}^{\left(3\right)},\cdots ,{\mathrm{DC}}_{m}^{\left(1\right)},{\mathrm{DC}}_{m}^{\left(2\right)},{\mathrm{DC}}_{m}^{\left(3\right)}\right)\;,\;$$

where ${\mathrm{DC}}_{j}^{\left(n\right)}$ is the *n*-th colour channel value of the *j*-th DC (j=1,⋯,m).

#### 4.2.2. Signed Surrounding Differences

**Figure 11:**
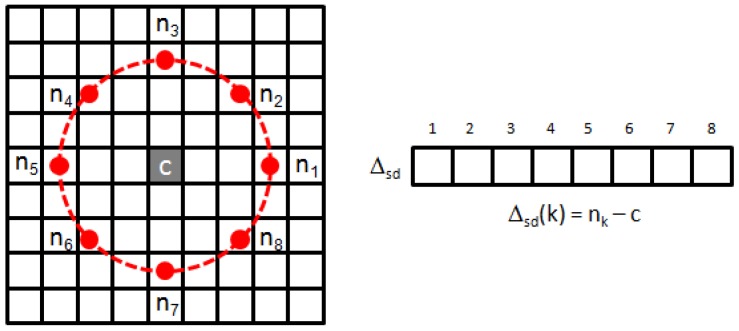
Illustration of *signed (surrounding) differences* $\Delta_{\mathrm{sd}}$ for p=8 and r=3.

The texture descriptor is built from statistical measures of the *signed (surrounding) differences* (SD) between a central pixel *c* and its *p* neighbours $n_{k}$ at a given radius *r*, similarly to the *local binary patterns* (LBP) first described by Ojala et al. [54], but keeping the magnitude of the difference (see Figure 11). Given colour channel *n*, the center-surround differences are calculated as follows:

(6) $$\Delta_{sd}^{\left(n\right)}\left(k\right)={\mathrm{bi}}^{\left(n\right)}\left(r\cos\alpha_{k},-r\sin\alpha_{k}\right)-c^{\left(n\right)}\;,\;\alpha_{k}=\frac{2\pi (k-1)}{p}\;,\;k=1,\cdots ,p$$

where ${\mathrm{bi}}^{\left(n\right)}\left(\cdot ,\cdot \right)$ refers to the approximation, by bilinear interpolation, of image point $n_{k}$ at the coordinates $\left(x,y\right)=\left(r\cos\alpha_{k},-r\sin\alpha_{k}\right)$ of colour plane *n*.

Next, given a patch of size ${\left(2w+1\right)}^{2}$ centered at the pixel under consideration, we account for the SD corresponding to all the pixels in the patch through a number of histograms: we employ different histograms for positive and for negative differences, and also for every colour channel, what makes necessary to calculate a total of six histograms per patch. Moreover, to counteract image noise (to a certain extent), our histograms group the SD into 32 bins; hence, since the maximum difference magnitude is 255 (in RGB space), the first bin accounts for magnitudes between 0 and 7, the second bin accounts for magnitudes between 8 and 15, etc. Finally, the texture descriptor consists of the *energies* of every histogram, i.e., sums of the corresponding squared probabilities Pr:

(7) $$\begin{array}{cc} D_{\mathrm{texture}} & =\left(\sum_{\Delta \geq 0}Pr{\left(\Delta_{sd}^{\left(1\right)}\right)}^{2},\sum_{\Delta \geq 0}Pr{\left(\Delta_{sd}^{\left(2\right)}\right)}^{2},\right.\sum_{\Delta \geq 0}Pr{\left(\Delta_{sd}^{\left(3\right)}\right)}^{2},\\ & \left.\sum_{\Delta <0}Pr{\left(\Delta_{sd}^{\left(1\right)}\right)}^{2},\sum_{\Delta <0}Pr{\left(\Delta_{sd}^{\left(2\right)}\right)}^{2},\sum_{\Delta <0}Pr{\left(\Delta_{sd}^{\left(3\right)}\right)}^{2}\right) \end{array}$$

Notice that the SD (Equation (6) and Figure 11) can be pre-calculated for every pixel of the full image. In this way, we can later compute the patch-level histograms, required to find the texture descriptor (Equation (7)), sharing the SD calculations among overlapping patches.

## 5. Experimental Results

In this section, we describe first the process followed to find an optimal configuration for the CBC detector, and compare it with other alternative combinations of colour and texture descriptors. Next, we report on the detection results obtained for some image sequences captured during flights inside a real vessel during a recent field trials campaign.

### 5.1. Configuration of the CBC Detector

To configure and assess the CBC detector, in this section we run a number of experiments involving a dataset comprising images of vessel structures affected, to a greater or lesser extent, by coating breakdown and different kinds of corrosion, and coming from several, different vessels and vessel areas, including those visited during the field trials mentioned above. Those images have been collected at different distances and under different lighting conditions. We refer to this dataset as the *generic corrosion* dataset. A hand-made ground truth has also been generated for every image involved in the assessment, in order to produce quantitative performance measures. The dataset, together with the ground truth, is available from [55]. Some examples of these images and the ground truth can be found in Figure 9.

To determine a sufficiently general configuration for the CBC detector, we consider variations in the following parameters:

- Half-patch size: *w* = 3, 5, 7, 9 and 11, giving rise to neighbourhood sizes ranging from 7×7=49 to 23×23=529 pixels.
- Number of DC: *m* = 2, 3 and 4.
- Number of neighbours *p* and radius *r* to compute the SD: (r,p)=(1,8) and (r,p)=(2,12).
- Number of neurons in the hidden layer: hn=⌈f×n⌉, with *f* = 0.6, 0.8, 1, 1.2, 1.4, 1.6, 1.8 and 2. Taking into account the previous configurations, the number of components in the input patterns *n* varies from 12 (m=2) to 18 (m=4), and hence hn goes from 8 (m=2, f=0.6) to 36 (m=4, f=2).

In all cases, all neurons make use of the hyperbolic tangent activation function with a=1 (see Section 4.1). In this way, the output of the neural network *o* is always a value between −1 and 1, respectively corresponding to the NC and the CBC classes. Typically, pattern $x_{i}$ should be classified as CBC if its output value $o_{i}$ is closer to 1 than to −1. To determine whether another approach would be beneficial, we consider a threshold τ∈[0,1) to classify the pattern as CBC ($o_{i}\geq \tau$) or NC ($o_{i}<\tau$). The final consequence of all these variations in the network parameters is a total of 5(patchsizes)×3(#DC required)×2(r-pcombinationsforSD)×8(#hidden neurons)=240 FFNNs to be trained and evaluated for 10 different threshold values *τ* = 0, 0.1, 0.2, 0.3, 0.4, 0.5, 0.6, 0.7, 0.8 and 0.9, leading to a total of 2400 assessments.

All configurations have been evaluated at the patch level using the same training and test sets (although *w* changes give rise to different patches, we ensure they all share the same center), which have been generated following the next rules:

1. We select a number of patches from the images belonging to the *generic corrosion* dataset. The set of patches is split into the *training patch set* and the *test patch set* (additional patches are used to define a *validation patch set*, which will be introduced later).
2. A patch is considered *positive* (CBC class) if the central pixel appears labelled as CBC in the ground truth. The patch is considered *negative* (NC class) if none of its pixels belong to the CBC class.
3. Positive samples are thus selected using ground truth CBC pixels as patch centers and shifting them a certain amount of pixels s<2w+1 to pick the next patch in order to ensure a certain overlapping between them (ranging from 57% to 87% taking into account all the patch sizes), and, hence, a rich enough dataset.
4. Negative patches, much more available in the input images, are selected randomly trying to ensure approximately the same number of positive and negative patterns, to prevent training from biasing towards one of the classes.
5. Initially, 80% of the set of patches are placed in the *training patch dataset*, and the remaining patches are left for testing.
6. Training, as far as the CBC class is concerned, is constrained to patches with at least 75% of pixels labelled as CBC. This has meant that, approximately, 25% of the initial training patches have had to be moved to the *test patch set*. Notice that this somehow penalizes the resulting detector during testing—i.e., consider the extreme case of a patch with only the central pixel belonging to the CBC class. In any case, it is considered useful to check the detector generality.

Additionally, following common good practices in machine learning, input patterns are normalized before training to avoid large dynamic, non-zero centered ranges in one dimension from affecting learning in other dimensions and thus favour quick convergence of the optimization algorithms involved in training [56]. Normalization is performed to ensure that all descriptor components lie in the interval [−0.95, +0.95]. Weight initialization is done following the Nguyen-Widrow method [57,58] so that the active regions of the hidden neurons are distributed approximately evenly over the input space. Finally, we make use of *iRprop* [59] to optimize the network weights. Table 1 summarizes the parameters of the optimizing algorithm as well as the main facts of the training and testing processes. *iRprop* parameters were set to the default values recommended by Igel and Hüsken in [59] since optimization was observed to progress adequately, i.e., reducing, without oscillations, the network error from iteration to iteration during training.

After training and evaluation (using the *test patch set*), *true positive rates* (TPR), *false positive rates* (FPR), and the *accuracy* metric (A) are calculated for the 2400 cases:

(8) $$TPR=\frac{TP}{TP+FN}\;,\;FPR=\frac{FP}{TN+FP}\;,\;A=\frac{TP+TN}{TP+TN+FP+FN}$$

where, as mentioned above, the *positive* label corresponds to the CBC class. Furthermore, given the particular nature of this classification problem, which is rather a case of *one-class classification*, i.e., detection of CBC against any other category, so that positive cases are clearly identified contrary to the negative cases, we also consider the harmonic mean of *precision* (P) and *recall* (R), also known as the *F1 measure* [60]:

(9) $$\begin{array}{cc} & P=\frac{TP}{TP+FP}\;,\;R=\frac{TP}{TP+FN}\;\left(=TPR\right) \end{array}$$

(10) $$\begin{array}{cc} & F1=\frac{2\;P\cdot R}{P+R}=\frac{2\;TP}{2\;TP+FP+FN} \end{array}$$

Notice that F1 values closer to 1 correspond to better classifiers.

**Figure 12:**
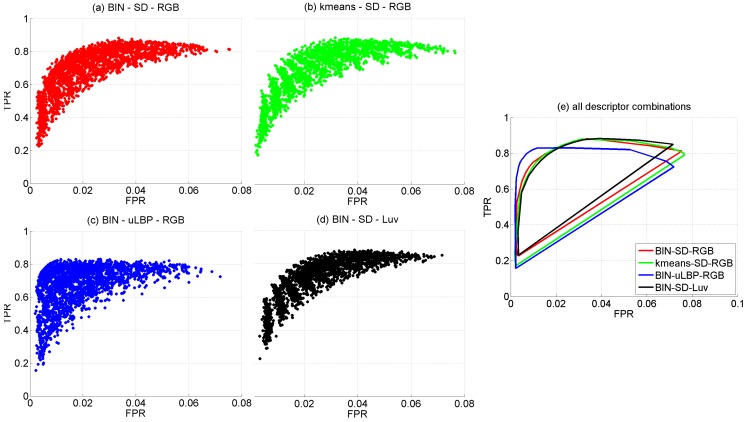
FPR *versus* TPR for all descriptor combinations: (**a**) BIN - SD - RGB; (**b**) k-means - SD - RGB; (**c**) BIN - uLBP - RGB; (**d**) BIN - SD - L^*^u^*^v^*^; (**e**) convex hulls of the FPR-TPR point clouds corresponding to each combination of descriptors.

Figure 12a plots in FPR-TPR space the full set of 2400 configurations of the CBC detector. Within this space, the perfect classifier corresponds to point (0,1). Consequently, among all classifiers, those whose performance lie closer to the (0,1) point are clearly preferrable to those ones that are farther, and hence distances to point (0,1) $d_{0,1}$ can also be used as a sort of performance metric. The same applies to the P-R space and point (1,1). Table 2a–d show, respectively, minimum $d_{0,1}$, maximum *accuracy*, minimum $d_{1,1}$ and maximum F1 values for every combination of the *w*, *r*, *p* and *m* parameter values. In all cases, the fraction of hidden neurons *f* and the value of threshold *τ* are indicated in parentheses. As can be observed, best configurations coincide in patch widths *w* of 9 and 11 pixels, (r,p) = (2,12) and 3 or 4 dominant colours (parameter *m*). According to Table 2a–d, absolute minimum $d_{0,1}/d_{1,1}$ and maximum *accuracy*/F1 values correspond to the same case: w=9, (r,p)=(2,12), m=3, hn=30 (input patterns consist of 6+3×3=15 components and the best value for *f* is 2) and τ=0. Other combinations employing smaller patches (what will reduce the execution time of both the colour and the texture descriptors), but leading to good performance, are (w=5,r=2,p=12,m=3,f=1.4/1.8) and (w=7,r=2,p=12,m=2,f=2).

In a second round of tests, the dominant colours have been determined making use of *k-means* [48], instead of the BIN method, with initial centers chosen by means of k-means++ [61]. k-means++ chooses carefully the initial seeds employed by k-means, in order to avoid poor clusterings. In essence, the algorithm chooses one center at random from among the patch colours; next, for each other colour, the distance to the nearest center is computed and a new center is selected with probability proportional to those distances; the process repeats until the desired number of DC is reached and k-means runs next. The seeding process essentially spreads the initial centers throughout the set of colours. This strategy has been proved to reduce the final clustering error as well as the number of iterations until convergence. Figure 12b plots the full set of configurations in FPR-TPR space. In this case, the minimum $d_{0,1}$/$d_{1,1}$ distances and the maximum A/F1 values are, respectively, 0.1242, 0.1243, 0.9222, 0.9219, slightly worse than the values obtained for the BIN method. All values coincide, as before, for the same configuration, which, in turn, is the same as for the BIN method. As can be observed, although the FPR-TPR plots are not identical, they are very similar. All this suggests that there are not many differences between the calculation of dominant colours by one (BIN) or the other method (k-means).

Analogously to the previous set of experiments, in a third round of tests, we change the way how the other part of the patch descriptor is built: we adopt stacked histograms of *uniform local binary patterns* (uLBP) [54] as texture descriptor, one histogram for every colour channel (similarly to the SD-based descriptor). LBPs code the intensity differences with surrounding pixels as 0-1 values (with 1 representing positive or null difference, and 0 for the negative case), and, hence, express local image texture through a compact, binary code of as many bits as neighbours. uLBPs group rotationally equivalent codes with zero or exactly two 0-1/1-0 transitions (one of each). As well as for SD, we consider p=8 and p=12 neighbours at, respectively, distances r=1 and r=2, and employ bilinear interpolation to approximate the pixel values. As well as in the work by Ojala et al. [54], we discard non-uniform codes. Consequently, 9 or 13-bin histograms result for the p=8 and p=12 cases, leading to, respectively, texture descriptors comprising 3×9=27 and 3×13=39 components. Figure 12c plots the full set of configurations in FPR-TPR space. In this case, the minimum $d_{0,1}$/$d_{1,1}$ distances and the maximum A/F1 values are, respectively, 0.1706, 0.1706, 0.9042, 0.9019, and are attained for w=11, r=1, p=8, m=2, f=1.2 and τ=0. The resulting performance is also worse than for the SD-based texture descriptor. Notice that, in general, SD are richer than LBPs, since the latter code the signs of the surrounding differences but not their magnitude, which may become relevant if contrast is one of the relevant features of the texture. Given the results obtained, it is clear that the SD-based descriptor, comprising both sign and magnitude of surrounding differences, is more adequate than the uLBP-based descriptor for this particular texture.

In a fourth and last round of tests, colour and texture data have been obtained by means of, respectively, the BIN method and the SD statistics, but both have been calculated over the CIE L^*^u^*^v^*^colour space, instead of over RGB. The CIE L^*^u^*^v^*^colour space is considered because of the well known properties of this space regarding perceptual colour differences, opposite to RGB, whose components are linearly related to primary luminances and readily available from the imaging sensor. The FPR-TPR plot with all configurations can be found in Figure 12d. In this case, the minimum $d_{0,1}$/$d_{1,1}$ distances and the maximum A/F1 values are, respectively, 0.1235, 0.1235, 0.9204 and 0.9203, not far from using RGB. The optimal configuration is not identical to the RGB case, although only parameter *f* changes, from 2 to 1.8.

**Figure 13:**
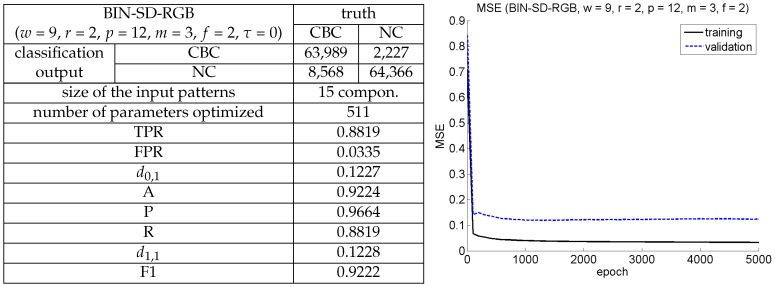
Best configuration of the CBC detector: (**Left**) performance details (*test patch set*); (**Right**) evolution of MSE during training, for the *training patch set* and the *validation patch set*.

Figure 12e superimposes the convex hulls of the FPR-TPR point clouds to make easier appreciate, from a more global perspective, the performance of the different combinations of colour and texture descriptors involved in the above-performed comparison. Additionally, Figure 13 provides relevant details of the training/learning processes for the BIN-SD-RGB best configuration, including the evolution of the *mean squared error* (MSE) during training for a total of 5000 epochs. As can be seen, the training error stabilizes more or less after 1000 epochs, while the error resulting for the *validation patch set* (a small fraction of additional patches from the *generic corrosion* dataset, not used for training nor for testing) does not increase significantly; no overfitting is thus observed.

**Figure 14:**
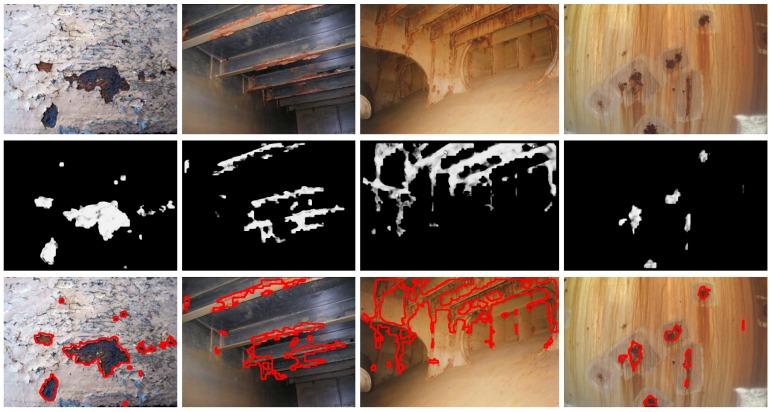
Examples of CBC detection for the *generic corrosion* dataset (I): (**Top**) original images; (**Middle**) CBC detector output; (**Bottom**) detection contours superimposed in red.

**Figure 15:**
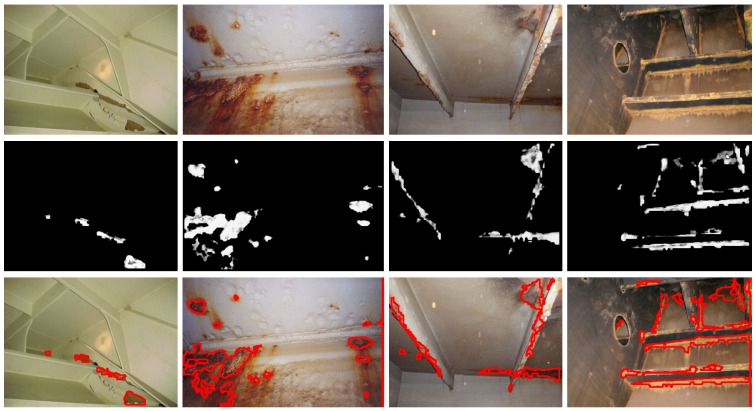
Examples of CBC detection for the *generic corrosion* dataset (II): (**Top**) Original images; (**Middle**) CBC detector output; (**Bottom**) Detection contours superimposed in red.

**Figure 16:**
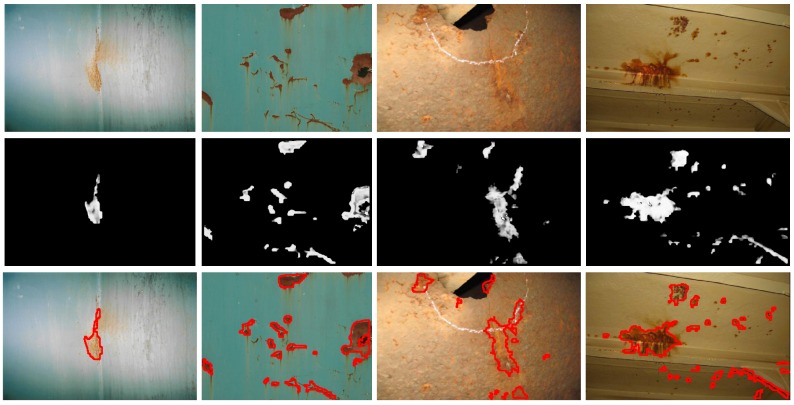
Examples of CBC detection for the *generic corrosion* dataset (III): (**Top**) Original images; (**Middle**) CBC detector output; (**Bottom**) Detection contours superimposed in red.

Figure 14, Figure 15 and Figure 16 show detection results at the pixel level for a selection of images of the *generic corrosion* dataset. In every figure, the middle row shows the output of the CBC detector: negative values are shown as black, while positive values are shown as shades of gray proportionally to the detector output (ranging from 0 to 1 for the positive side). The bottom row shows the contours of the resulting regions superimposed over the original image. To obtain these results, every image has been processed patch by patch, setting their centers at the points of a grid with step s≤2w+1. In case the patch center is classified as CBC by the detector (i.e., the NN output is greater or equal than τ=0), every pixel of the patch is also explored to determine whether it also belongs to the CBC class or not and produce a finer detection. If the center does not belong to the CBC class, no other pixel of the patch is considered and the search continues in the next patch, whose center will be located in the next grid point. Once all the image pixels have been considered and NN outputs are available for them, a final post-processing step follows, in which those outputs are median-filtered using a 3×3 support region.

**Figure 17:**
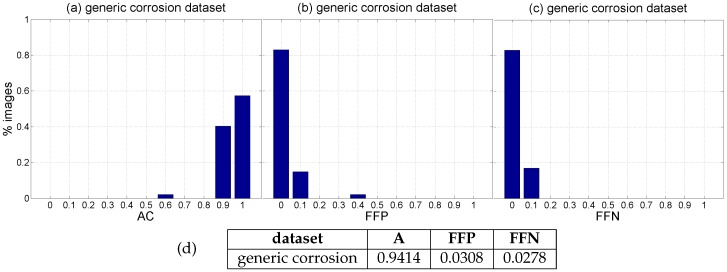
Global performance histograms, at the pixel level, for the *generic corrosion* dataset: (**a**) *Accuracy* values; (**b**) *Fraction of false positives*; (**c**) *Fraction of false negatives*; (**d**) Average performance values.

Finally, global performance data for every image of the *generic corrosion* dataset can be found in Figure 17. To this end, TP, TN, FP and FN have been evaluated at the pixel level. This makes dramatically relevant deviations of just one pixel right, left, up and/or down while generating the ground truth (what is relatively likely). In order to counteract to a certain extent this excessive influence, we consider correct those classifications for which the distance between a positive prediction and a positive in the ground truth is less than or equal to 5 pixels. Besides, it must be noticed that most of the typical metrics used to evaluate the classification output, i.e., Equations (8)–(10), can become undefined for a given image because one or several of the respective quotients become zero. This is because an image is not a dataset which purposively contains samples from all the classes involved in the classification problem. By way of example, consider the extreme case of an image which does not contain any pixel affected by corrosion, which in turn is classified correctly by the defect detector; this situation makes zero all the quotients of Equations (8)–(10) except for the *accuracy* metric, since TN = “all image pixels” and consequently TP = 0, FP = 0 and FN = 0. Other cases which make zero one or several of those quotients typically arise when there is “nothing or almost nothing to detect” in the image. Because of the aforementioned, to show global performance at the pixel level, Figure 17a plots a histogram of *accuracy* values, which provides information about correct classifications, while Figure 17b,c respectively plot histograms of the *fraction of false positives* (FFP) and the *fraction of false negatives* (FFN), i.e., the classification errors:

(11) $$FFP=\frac{FP}{TP+TN+FP+FN}\;,\;FFN=\frac{FN}{TP+TN+FP+FN}$$

Average performance values for this dataset can be found in Figure 17d. (Notice that the *accuracy* metric is equivalent to the *discrepancy percentage* [62,63], a metric for evaluating image segmentation results.)

Summing up, taking into account the quantitative and qualitative performance data reported for the *generic corrosion* dataset, we can say:

1. Regarding the *patch test set*, TPR = R = 0.8819 and FPR = 0.0335 respectively indicate that less than 12% of positive patches and around 3% of negative patches of the set are not identified as such, while A = 0.9224 means that the erroneous identifications represent less than 8% of the total set of patches.
2. At the pixel level, A = 0.9414, i.e., *accuracy* turns out to be higher than for patches, leading to an average incidence of errors (1 − A = FFP + FFN) of about 5%, slightly higher for false positives, 3.08% against 2.78%.
3. Figure 14, Figure 15 and Figure 16, reporting on defect detection performance at a qualitative level, show accurate CBC detection.
4. In accordance to the aforementioned, the CBC detector can be said to perform well under general conditions, improving at the pixel level (5% of erroneous identifications) against the *test patch set* (8% of erroneous identifications).

### 5.2. Results for Field Test Images

**Figure 18:**
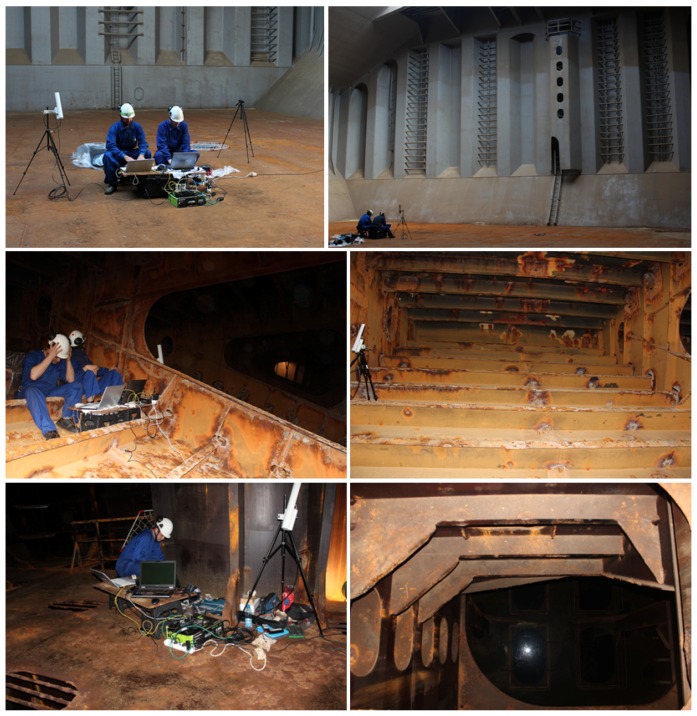
Some pictures about the tests performed inside the bulk carrier: (**Top**) cargo hold; (**Middle**) top-side tank; (**Bottom**) fore-peak tank.

**Figure 19:**
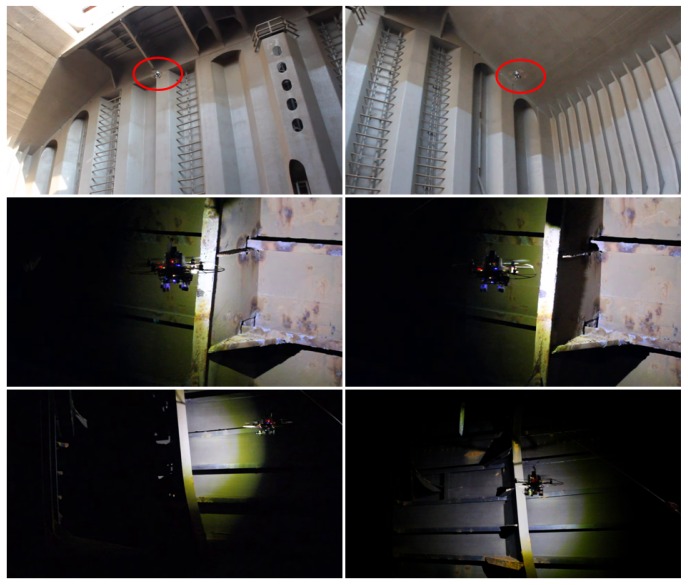
Pictures from some of the flights performed inside the bulk carrier: (**Top**) cargo hold; (**Middle**) top-side tank; (**Bottom**) fore-peak tank.

**Figure 20:**
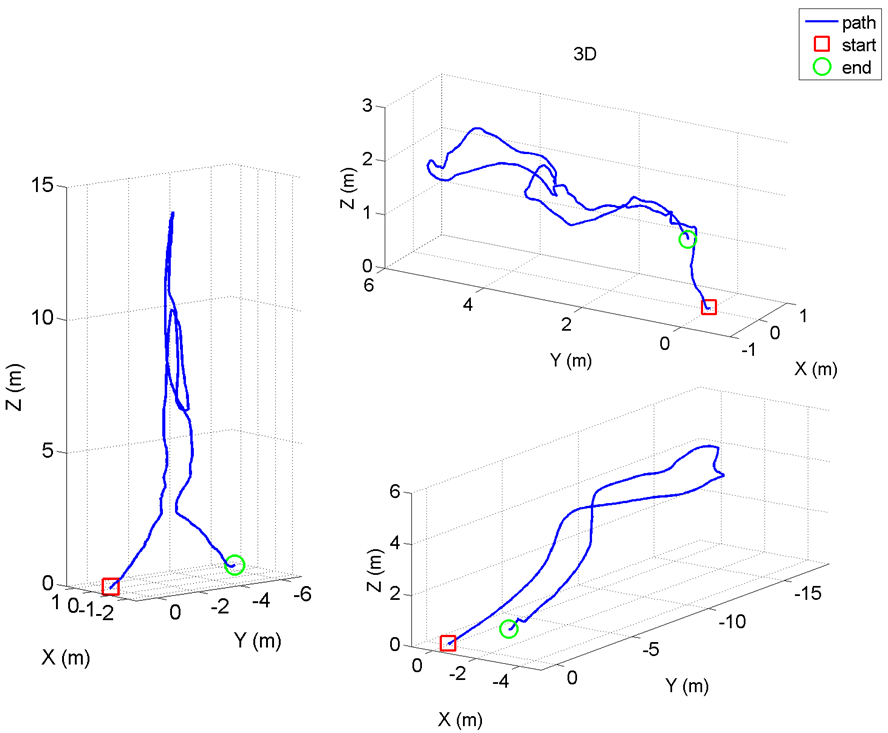
Trajectories estimated for some of the flights performed inside the bulk carrier.

This section reports on the results obtained for a number of images captured during a campaign of field experiments taking place onboard a 50.000 DWT bulk carrier while at port in May 2016. Images were captured during real flights within several scenarios of the vessel, taking advantage of the many features implemented in the MAV control architecture oriented towards improving image quality and, ultimately, defect detection performance. In more detail, the MAV was flown inside one of the cargo holds, in open-air, and also within the fore-peak tank and within one of the top-side ballast tanks, fitted both places with a single, manhole-sized entry point and limited visibility without artificial lighting. Some pictures about the tests in the different environments can be found in Figure 18 and Figure 19. Videos about the trials are available from [64] (cargo hold), from [65] (top-side tank), and from [66] (fore-peak tank). By way of example, Figure 20 plots the trajectories estimated for some of the flights performed during the inspections.

More than 200 images from the aforementioned environments captured during some of those flights have been selected for an additional evaluation of the CBC detector under flying conditions. These images define the *cargo hold*, *top-side tank* and *fore-peak tank* datasets which we will refer to in this section, comprising thus images coming from exclusively flights performed with the MAV described in Section 3. Ground truth data has also been generated for all those images, in order to obtain quantitative performance assessments, as in Section 5.1. (A fraction of these images are also part of the *generic corrosion* dataset we make use of in Section 5.1, representing, in that case, images from one among several vessels/vessel areas this dataset consists of.)

**Figure 21:**
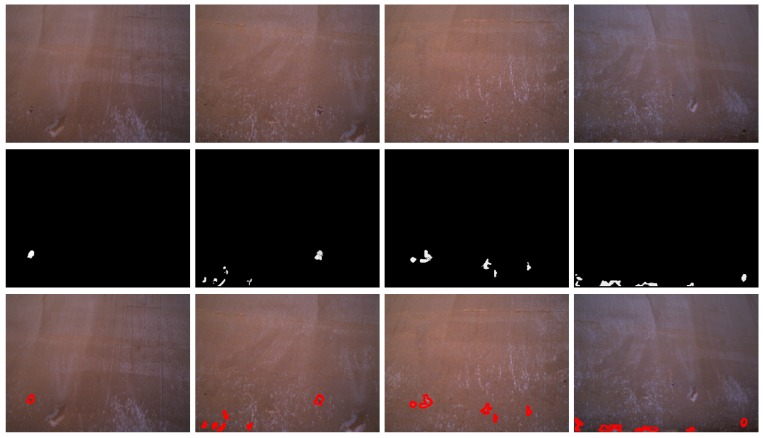
Examples of CBC detection for the *cargo hold* dataset (I): (**Top**) original images; (**Middle**) CBC detector output; (**Bottom**) detection contours superimposed in red.

**Figure 22:**
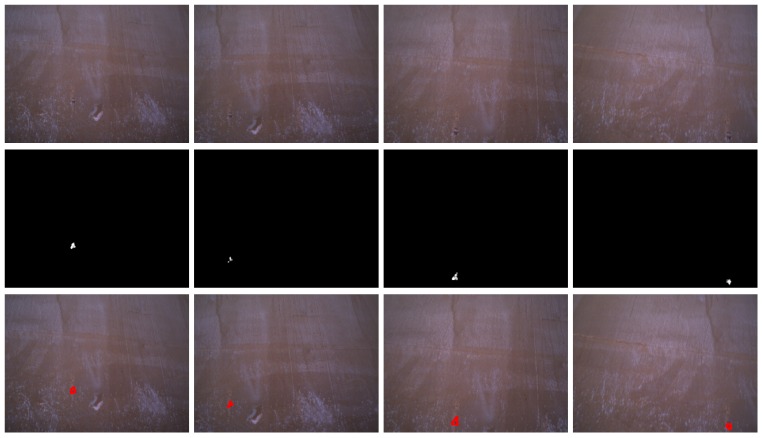
Examples of CBC detection for the *cargo hold* dataset (II): (**Top**) original images; (**Middle**) CBC detector output; (**Bottom**) detection contours superimposed in red.

**Figure 23:**
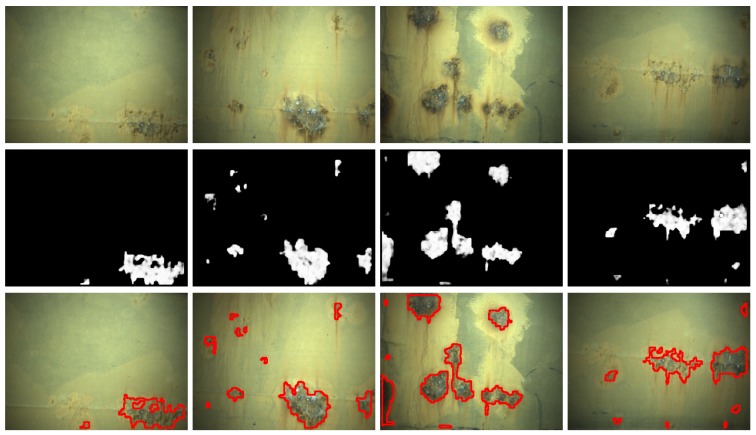
Examples of CBC detection for the *top-side tank* dataset (I): (**Top**) original images; (**Middle**) CBC detector output; (**Bottom**) detection contours superimposed in red.

**Figure 24:**
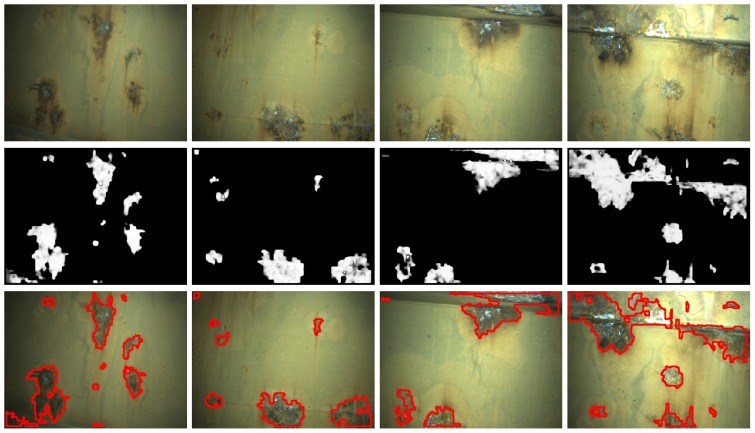
Examples of CBC detection for the *top-side tank* dataset (II): (**Top**) original images; (**Middle**) CBC detector output; (**Bottom**) detection contours superimposed in red.

**Figure 25:**
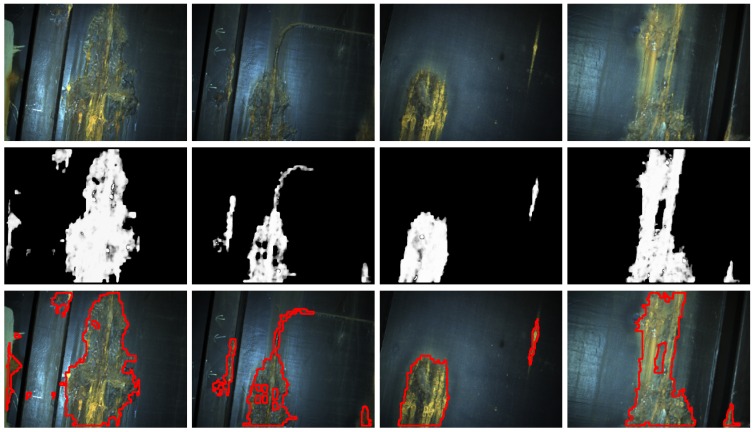
Examples of CBC detection for the *fore-peak tank* dataset (I): (**Top**) original images; (**Middle**) CBC detector output; (**Bottom**) detection contours superimposed in red.

**Figure 26:**
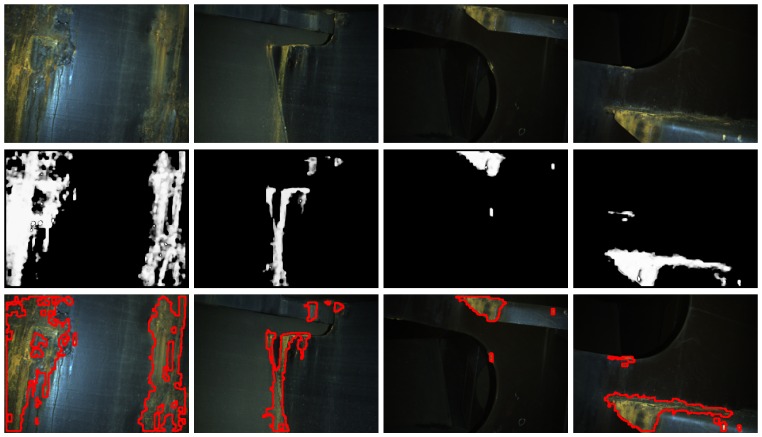
Examples of CBC detection for the *fore-peak tank* dataset (II): (**Top**) original images; (**Middle**) CBC detector output; (**Bottom**) detection contours superimposed in red.

**Figure 27:**
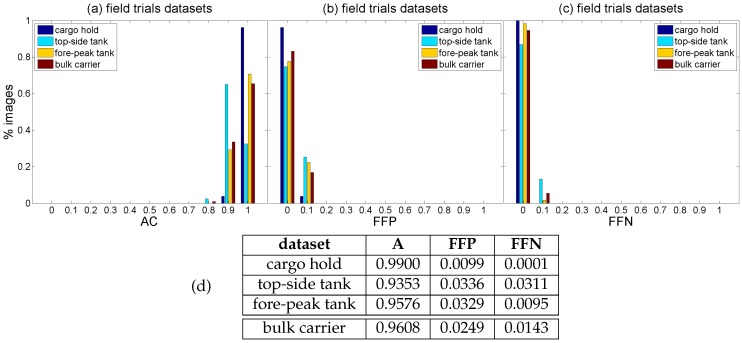
Global performance histograms, at the pixel level, for the *cargo hold*, *top-side tank* and *fore-peak tank* datasets alone and jointly for the whole vessel: (**a**) *Accuracy* values; (**b**) *Fraction of false positives*; (**c**) *Fraction of false negatives*; (**d**) Average performance values.

Figure 21 and Figure 22 show detection results for some of the images captured during the flights inside the cargo gold. This area of the vessel was in pretty good condition, so that not many CBC detections could be expected, as can be seen from the results obtained. The other two areas of the vessel did contain a number of cases of CBC, as can be observed from Figure 23 and Figure 24 for the top-side tank and Figure 25 and Figure 26 for the fore-peak tank. As mentioned above, both areas are usually not illuminated, what required the activation of the MAV spotlight during flight. Global performance results for the field trials, i.e., considering all three datasets alone and jointly for the whole vessel, are shown in Figure 27a–c in the form of, respectively, histograms of *accuracy* values, *fraction of false positives* and *fraction of false negatives*, in the same way it has been done for the *generic corrosion* dataset. Average values can be found in Figure 27d.

As can be observed, classification performance is slightly better than the one obtained for the *generic corrosion* dataset, with the CBC detector behaving well in general for the three datasets/environments, with a similar, low amount of classification errors representing on average around 3% of the image pixels, once again slightly higher regarding false positives.

### 5.3. Some Comments on the Time Complexity of the Defect Detector

Regarding the time complexity of the classifier, most part of the time required is spent on computing the patch descriptor. As mentioned before, the SD are pre-computed at the pixel level for all the image; next, the statistics expressed in Equation (7) are calculated at the patch level, sharing the computation of the SD for the pixels belonging to overlapping patches. The calculation of the SD is of the order of the number of neighbours (*p*) and the size of the image (V×H pixels), while the computation time of the SD statistics depends on the size of the patch (${\left(2w+1\right)}^{2}$) and on the number of bins of the SD histograms (set to 32). As for the DC, they must be calculated directly at the patch level, so no pre-calculation is possible. The DC are determined through an iterative process, with as many iterations as the number of DC (*m*). At every iteration, all pixels of the patch are considered, so time complexity depends on the patch size (${\left(2w+1\right)}^{2}$).

**Figure 28:**
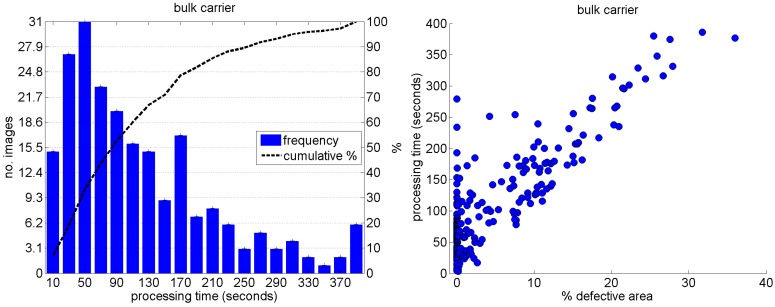
Processing times for the *cargo hold*, *top-side tank* and *fore-peak tank* datasets: (**Left**) histogram; (**Right**) processing time *versus* percentage of defective area in the image.

Besides, as explained in Section 5.1, in case the patch center is classified as CBC by the detector, every pixel of the patch is also explored to determine whether it also belongs to the CBC class or not and produce a finer detection. This means that the processing time depends on the number and size of the defects appearing in an image. On most occasions, images do not contain any or very few defects, so lower execution times are likelier. This can be observed in the histogram of Figure 28 (left), which accounts for the processing times corresponding to the images of the *cargo hold*, *top-side tank* and *fore-peak tank* datasets, and also in the plot of Figure 28 (right), which shows the relationship between the percentage of defective area in the image (according to the ground truth) and the processing time. We choose these datasets because they all come from the Pointgrey camera mentioned in Section 3.1 and hence have the same size, contrary to the case of the images of the *generic corrosion* dataset.

All times correspond to an Intel Core i7 processor fitted with 32Gb of RAM and running Windows 10. Hence, some increments of the execution time which can be observed in Figure 28 can be attributed to sporadic overhead from the operating system, such as those cases of Figure 28 (right) which detach from the apparently linear relationship between percentage of defective area and execution time. Besides, it is also important to note that, apart from the pre-computation of the SD, no other optimization has been incorporated in the code to reduce the processing time. It is left as future work adopting speedup strategies, such as multithreading, use of Intel processors’ SIMD instructions, and/or use of GPGPU units. In any case, apart from the fact that reducing the execution time is interesting per se, it must be noticed that this application does not involve any requirement of real-time operation.

## 6. Conclusions

An approach for *coating breakdown/corrosion* (CBC) detection in vessel structures has been described. It comprises (1) a semi-autonomous MAV fitted with functionalities intended to enhance image capture by means of extensive use of behaviour-based high-level control; and (2) a neural network to detect pixels belonging to CBC-affected areas. Classification is performed on the basis of the neighbourhood of every image pixel, computing a descriptor that integrates both colour and texture information. Colour data is supplied in the form of *dominant colours* (DC), while texture data is based on statistics of *signed (surrounding) differences* (SD). Both kinds of information are obtained directly from the RGB image. Successful detection results have been reported for a generic and varied image set comprising coating breakdown and corrosion defects, which has been in turn used to design and tune the CBC detector, as well as to compare the proposed descriptors with alternative ways of describing colour and texture. Besides, we have provided detection results for images captured by the MAV during a number of flights performed inside a bulk carrier in the framework of a campaign of field tests taking place in 2016.

Future enhancement steps comprise the fusion of the laser scanner with optical-flow sensors, in order to enlarge the number of environments which can be inspected: i.e., optical-flow sensors get in trouble at inadequately illuminated scenarios, where the laser scanner succeeds, while laser scanners can become not so useful in well-lit areas but without enough structure that permits to produce reliable laser-scan matchings. Regarding the CBC detector, speeding up strategies, either using multithreading, Intel processors’ SIMD instructions, and/or GPGPU units, are under consideration, as well as a stronger integration between the onboard range sensors and feature extraction to further increase defect detection performance (beyond the support provided by the platform control software) through, e.g., scale and viewpoint normalization.

## Figures and Tables

**Table 1:** Training/testing parameters (see [59] for an explanation of the *iRprop* parameters).

Parameter	Symbol	Value
activation function free parameter	*a*	1
iRprop weight change increase factor	$\eta^{+}$	1.2
iRprop weight change decrease factor	$\eta^{-}$	0.5
iRprop minimum weight change	$\Delta_{\min}$	0
iRprop maximum weight change	$\Delta_{\max}$	50
iRprop initial weight change	$\Delta_{0}$	0.5
(final) number of training patches		232,094
— positive patches		120,499
— negative patches		111,595
(final) number of test patches		139,150
— positive patches		72,557
— negative patches		66,593

**Table 2:** CBC detector performance data [BIN-SD-RGB combination]: (a) minimum distance to the (0,1) point in FPR-TPR space; (b) *accuracy* values; (c) minimum distance to the (1,1) point in P-R space; (d) F1 values. Values in parentheses are the corresponding *f* and *τ* values. Best global configuration is highlighted in bold.

(a)		*w*				
(r,p)	*m*	3	5	7	9	11
	2	0.1747 (1.0,0.0)	0.1688 (1.8,0.0)	0.1512 (1.6,0.0)	0.1484 (1.2,0.0)	0.1629 (1.8,0.0)
(1, 8)	3	0.1712 (2.0,0.0)	0.1600 (1.4,0.0)	0.1496 (1.4,0.0)	0.1382 (1.6,0.0)	0.1692 (1.8,0.0)
	4	0.1696 (2.0,0.0)	0.1741 (2.0,0.0)	0.1657 (1.6,0.0)	0.1501 (2.0,0.0)	0.1795 (1.8,0.0)
	2	0.1849 (1.6,0.0)	0.1627 (1.6,0.0)	0.1377 (2.0,0.0)	0.1370 (2.0,0.0)	0.1574 (1.2,0.0)
(2,12)	3	0.1652 (2.0,0.0)	0.1519 (1.8,0.0)	0.1450 (1.4,0.0)	**0.1227** (2.0,0.0)	0.1468 (2.0,0.0)
	4	0.1641 (1.8,0.0)	0.1555 (1.2,0.0)	0.1473 (0.8,0.0)	0.1394 (1.2,0.0)	0.1735 (0.6,0.0)
(b)		*w*				
(r,p)	*m*	3	5	7	9	11
	2	0.8851 (1.0,0.0)	0.8908 (1.8,0.0)	0.9027 (1.6,0.0)	0.9050 (1.2,0.0)	0.9004 (1.8,0.0)
(1, 8)	3	0.8873 (2.0,0.0)	0.8956 (1.4,0.0)	0.9034 (1.4,0.0)	0.9124 (1.6,0.0)	0.8964 (1.8,0.0)
	4	0.8881 (2.0,0.0)	0.8886 (2.0,0.0)	0.8965 (1.6,0.0)	0.9075 (2.0,0.0)	0.8930 (1.8,0.0)
	2	0.8800 (1.6,0.0)	0.8941 (2.0,0.0)	0.9116 (2.0,0.0)	0.9142 (2.0,0.0)	0.9024 (1.2,0.0)
(2,12)	3	0.8915 (2.0,0.0)	0.9010 (1.4,0.0)	0.9070 (1.8,0.0)	**0.9224** (2.0,0.0)	0.9107 (2.0,0.0)
	4	0.8928 (1.8,0.0)	0.9003 (1.2,0.0)	0.9055 (1.4,0.0)	0.9135 (1.6,0.0)	0.8980 (2.0,0.0)
(c)		*w*				
(r,p)	*m*	3	5	7	9	11
	2	0.1753 (1.0,0.0)	0.1694 (1.8,0.0)	0.1515 (1.6,0.0)	0.1487 (1.2,0.0)	0.1631 (1.8,0.0)
(1, 8)	3	0.1717 (2.0,0.0)	0.1603 (1.4,0.0)	0.1499 (1.4,0.0)	0.1383 (1.6,0.0)	0.1694 (1.8,0.0)
	4	0.1701 (2.0,0.0)	0.1748 (2.0,0.0)	0.1662 (1.6,0.0)	0.1504 (2.0,0.0)	0.1797 (1.8,0.0)
	2	0.1858 (1.6,0.0)	0.1631 (1.6,0.0)	0.1379 (2.0,0.0)	0.1372 (2.0,0.0)	0.1575 (1.2,0.0)
(2,12)	3	0.1656 (2.0,0.0)	0.1522 (1.8,0.0)	0.1452 (1.4,0.0)	**0.1228** (2.0,0.0)	0.1468 (2.0,0.0)
	4	0.1645 (1.8,0.0)	0.1558 (1.2,0.0)	0.1475 (0.8,0.0)	0.1396 (1.2,0.0)	0.1737 (0.6,0.0)
(d)		*w*				
(r,p)	*m*	3	5	7	9	11
	2	0.8837 (1.0,0.0)	0.8891 (1.8,0.0)	0.9017 (1.6,0.0)	0.9041 (1.2,0.0)	0.8995 (1.8,0.0)
(1, 8)	3	0.8860 (2.0,0.0)	0.8946 (1.4,0.0)	0.9025 (1.4,0.0)	0.9117 (1.6,0.0)	0.8953 (1.8,0.0)
	4	0.8870 (2.0,0.0)	0.8864 (2.0,0.0)	0.8942 (1.6,0.0)	0.9059 (2.0,0.0)	0.8908 (1.8,0.0)
	2	0.8776 (1.6,0.0)	0.8923 (1.6,0.0)	0.9111 (2.0,0.0)	0.9134 (2.0,0.0)	0.9020 (1.2,0.0)
(2,12)	3	0.8904 (2.0,0.0)	0.9003 (1.4,0.0)	0.9059 (1.8,0.0)	**0.9222** (2.0,0.0)	0.9105 (2.0,0.0)
	4	0.8916 (1.8,0.0)	0.8991 (1.2,0.0)	0.9044 (0.8,0.0)	0.9124 (1.6,0.0)	0.8959 (2.0,0.0)

## Abbreviations

The following main abbreviations are used in this manuscript:

A	Accuracy
CBC	Coating-Breakdown/Corrosion
DC	Dominant Colours
FFN	Fraction of False Negatives
FFP	Fraction of False Positives
FN	False Negatives
FP	False Positives
FPR	False Positive Rate
P	Precision
R	Recall
SD	Signed (surrounding) Differences
TN	True Negatives
TP	True Positives
TPR	True Positive Rate
